# Toward Standardized Platelet-Rich Plasma Therapy in Tendon Healing: Integrating Biological Characterization with Clinical Translation

**DOI:** 10.3390/ijms27167393

**Published:** 2026-08-18

**Authors:** Jeries Issa Alghishan, Bogdan Andor

**Affiliations:** 1 Department of Orthopedics and Traumatology, Victor Babeș University of Medicine and Pharmacy, 300723 Timișoara, Romania; 2 Teodor Șora Research Center, Victor Babeș University of Medicine and Pharmacy, 300723 Timișoara, Romania

**Keywords:** platelet-rich plasma, tendon healing, tendinopathy, regenerative medicine, growth factors, cytokines, platelet activation, biological characterization, imaging biomarkers, precision medicine

## Abstract

Platelet-rich plasma (PRP) has emerged as one of the most extensively investigated orthobiologic therapies for tendon disorders because of its potential to modulate inflammation, enhance extracellular matrix remodeling, and promote tissue regeneration through the delivery of concentrated platelets and bioactive molecules. However, despite compelling biological rationale and encouraging preclinical evidence, clinical outcomes remain inconsistent across different tendon pathologies. This narrative review critically examines the principal biological and methodological factors underlying this variability, including differences in cellular composition, growth factor and cytokine profiles, activation strategies, and current PRP classification systems. We further synthesize the available clinical evidence across major tendon disorders, highlighting the influence of disease-specific biology, product heterogeneity, and procedural variability on treatment response. In addition, the emerging role of quantitative imaging biomarkers in objectively evaluating tendon regeneration is discussed as a complementary tool for biological outcome assessment. Based on the evidence reviewed, we propose the quantifiable platelet-rich plasma (Q-PRP) framework, a practical reporting model that integrates cellular, molecular, procedural, and clinical variables into a standardized approach for biologically meaningful PRP characterization. Rather than replacing existing classification systems, the proposed framework aims to improve reproducibility, facilitate cross-study comparison, and support the transition toward precision regenerative medicine. Standardized biological characterization, combined with objective outcome assessment, may represent a critical step toward optimizing PRP research and clinical application in tendon healing.

## 1. Introduction

Tendon disorders represent one of the most prevalent causes of chronic musculoskeletal pain and long-term functional limitation in both athletic and general populations. Chronic musculoskeletal pain as a whole affects an estimated 20–33% of the global population, corresponding to more than one billion individuals, with women, older adults, and socioeconomically vulnerable groups disproportionately represented [1]. Low back pain and knee pain are the dominant clinical presentations, and they are consistently accompanied by functional decline, depressive symptoms and a measurable reduction in quality of life [1]. Within this broader landscape, tendinopathies of the Achilles tendon, patellar tendon, rotator cuff and lateral epicondyle constitute a substantial share of the clinical burden, particularly among middle-aged recreational athletes and workers engaged in repetitive overhead or load-bearing tasks [2].

Conventional management of tendinopathy—including activity modification, physiotherapy, non-steroidal anti-inflammatory drugs (NSAIDs), corticosteroids and, in refractory cases, surgery—provides symptomatic relief but does not restore the underlying tendon matrix. Prolonged NSAID use carries well-documented risks of gastrointestinal ulceration, renal injury, and adverse cardiovascular events, while opioid analgesics remain controversial because of their addiction potential and overdose-related mortality [1]. These limitations have motivated a sustained interest in regenerative biologic therapies that aim to enhance intrinsic tissue repair rather than solely suppressing inflammation. Among such therapies, platelet-rich plasma (PRP) has become the most widely investigated autologous strategy in orthopedics and sports medicine [1,2].

PRP is an autologous plasma fraction in which the platelet concentration exceeds that of whole blood, typically by a factor of two to six [1]. Upon activation, platelets release growth factors and cytokines from their α-granules, including the platelet-derived growth factor (PDGF), the transforming growth factor-β1 (TGF-β1), the vascular endothelial growth factor (VEGF), the basic fibroblast growth factor (bFGF), the epidermal growth factor (EGF), the insulin-like growth factor-1 (IGF-1), and the hepatocyte growth factor (HGF) [2,3]. The working hypothesis is that delivering these bioactive molecules at supraphysiological concentrations to a damaged tendon accelerates and improves the natural healing cascade by stimulating tenocyte proliferation, collagen synthesis, angiogenesis, and extracellular matrix remodeling [2].

Clinical translation of this hypothesis has, however, been uneven. Current clinical evidence suggests that PRP may provide clinical benefit in selected tendon disorders, including lateral epicondylitis and patellar tendinopathy; however, available evidence does not establish consistent superiority of leukocyte-rich PRP (LR-PRP) over leukocyte-poor PRP (LP-PRP), and the optimal cellular formulation remains uncertain and may be disease-specific [3,4,5]. The breadth of PRP use is nevertheless remarkable: an estimated 86,000 athletes in Europe and the United States receive PRP injections for tendon, ligament, or muscle injuries each year [6]. This discrepancy between widespread clinical adoption and inconsistent evidence has positioned PRP as both a promising regenerative tool and a controversial indication in tendon care [1,2].

A central explanation for the variable clinical performance of PRP lies in the heterogeneity of the products themselves. PRP is operationally defined, and two preparations bearing the same name can differ substantially in platelet concentration, leukocyte content, red blood cell contamination, activation strategy, and final volume [2,7]. Importantly, LR-PRP delivers elevated leukocyte—especially neutrophil—concentrations that are associated with higher levels of pro-inflammatory and catabolic mediators, including interleukin-1β (IL-1β), tumor necrosis factor-α (TNF-α), and matrix metalloproteinases (MMPs), which may antagonize the anabolic effects of PDGF at the injection site [7].

Compounding this biologic variability is a persistent deficit in reporting. A benchmark platelet concentration of 1 million/μL in a 6 mL aliquot (although this threshold remains largely empirical and has not been universally validated) is widely cited as the threshold for “therapeutic PRP”, yet most published studies report only the commercial kit used and omit the actual platelet yield, leukocyte count, red blood cell contamination and activation conditions of the administered product [6]. The result is a literature in which apparently comparable trials in fact compare products that may differ by an order of magnitude in cellular composition and growth factor content [2,8]. This unsolved heterogeneity is the principal obstacle to evidence-based optimization of PRP in tendon healing and the motivation for the present review.

The purpose of this review is to integrate current knowledge on PRP biology, classification, and clinical application in tendon disorders into a single, structured framework that explicitly links cellular composition, growth factor signature, and clinical outcome. Specifically, we (i) dissect why PRP outcomes in tendon disorders remain inconsistent across trials; (ii) critically appraise existing classification systems for PRP; (iii) summarize the cellular and molecular determinants of PRP activity, including platelet dose, leukocyte content, red blood cell contamination and growth factor release kinetics; (iv) review the evidence in major tendinopathies; (v) discuss imaging biomarkers of treatment response; and (vi) introduce the quantifiable platelet-rich plasma (Q-PRP) framework—a practical proposal for the minimum reporting variables and the quantitative descriptors that should accompany every future tendon–PRP study, in order to enable cross-study comparison, dose–response modeling, and clinical translation [8,9].

### Literature Search Strategy

A structured literature search was conducted to support this narrative review using PubMed/MEDLINE and Web of Science. The search included publications available up to August 2026, with no restriction on publication date. Search terms were combined according to the topics addressed in the review and included “platelet-rich plasma”, “PRP”, “tendon”, “tendinopathy”, “tendon healing”, “platelet concentration”, “platelet dose”, “leukocytes”, “growth factors”, “cytokines”, “activation”, “ultrasound”, “magnetic resonance imaging”, “clinical outcomes”, “classification”, and “standardization”. Original experimental and clinical studies, randomized controlled trials, systematic reviews, meta-analyses, consensus statements, and relevant classification or reporting frameworks were prioritized. Publications were selected according to their relevance to PRP composition, biological mechanisms, tendon healing, clinical efficacy, imaging assessment, and methodological standardization. As this work was designed as a narrative rather than a systematic review, the literature was synthesized thematically to address the mechanistic, clinical, and methodological objectives of the manuscript.

## 2. Why Are PRP Outcomes So Inconsistent?

PRP should not be considered a single standardized therapeutic product, but rather a heterogeneous group of autologous preparations whose biological properties depend on both patient-related and processing-related factors. Differences in baseline hematological characteristics, platelet and leukocyte content, preparation protocols, activation strategies, and administered dose can substantially alter the final product and complicate comparisons across studies [5,6,8,10].

Preparation protocols represent a major source of inter-study variability. Commercial and laboratory-based systems differ in blood volume, centrifugation conditions, platelet recovery, cellular purity, final PRP volume, and activation strategy, producing preparations with substantially different biological characteristics despite being reported under the same generic term “PRP” [8,9,10].

An additional source of heterogeneity is the inconsistent reporting of platelet exposure. Platelet concentration alone does not define the amount of platelets delivered to the target tissue, yet total platelet dose is frequently omitted from clinical studies, limiting interpretation of dose–response relationships [5,10,11,12]. This distinction is discussed in detail in Section 4.1.

Heterogeneity also arises from the treated pathology itself. Tendon disorders differ in anatomical environment, vascularity, mechanical loading, chronicity, and underlying tissue degeneration; therefore, a PRP formulation effective in one tendon disorder cannot be assumed to produce an equivalent response in another. Disease-specific clinical evidence is considered separately in Section 8 [3,4,5].

Leukocyte composition represents another major source of biological variability. The conventional distinction between LR-PRP and LP-PRP does not fully capture differences in leukocyte subsets, which may exert distinct inflammatory and regenerative effects. Consequently, both total leukocyte content and differential composition should be considered when interpreting PRP studies [6,13,14,15,16].

Taken together, these sources of variability demonstrate why the generic term “PRP” is insufficient to define the biological intervention administered in clinical studies. Meaningful interpretation of PRP outcomes therefore requires standardized characterization of both the product and the context in which it is delivered. Existing classification and reporting systems developed to address this problem are discussed in the following section [5,6,8,10].

## 3. Current Classification Systems for PRP

The recognition that PRP does not represent a single biologic entity but rather a spectrum of preparations has led to the development of several classification and reporting frameworks in orthopedics and sports medicine. Among them, the platelets, activation, white blood cells (PAW) system; the dose, efficiency, purity, activation (DEPA) classification; the method, activation, red blood cells, spin, platelets, image guidance, leukocytes, light activation (MARSPILL) taxonomy; and the Minimum Information for Studies Evaluating Biologics in Orthopedics (MIBO) recommendations are the most frequently discussed approaches. Although each framework was designed to facilitate comparison among studies and improve standardization, they differ substantially in scope, level of detail, and practical adoption.

### 3.1. PAW Classification

The PAW classification, originally proposed by DeLong and colleagues, characterizes PRP according to three independent dimensions: platelet concentration, activation status, and white blood cell content [6]. Bibliometric analyses indicate that PAW remains one of the most widely cited PRP classification systems in the tendon and ligament literature [6].

A major strength of PAW is the separate characterization of platelet concentration, activation strategy, and leukocyte composition, including neutrophil content, thereby distinguishing biologically relevant features that may otherwise remain hidden under the generic designation of PRP [6].

### 3.2. DEPA Classification

The DEPA classification evaluates PRP preparations according to four variables: platelet dose, platelet recovery efficiency, cellular purity, and activation characteristics. By integrating these parameters into a single scoring system, DEPA was conceived as both a classification tool and a quality-control framework for point-of-care devices.

DEPA introduced two particularly relevant quantitative concepts—platelet recovery efficiency and cellular purity—while retaining platelet dose and activation as core descriptors. Its principal limitation is the use of ordinal categories, which may compress biologically meaningful differences between preparations [8,9].

### 3.3. MARSPILL Classification

The MARSPILL framework was proposed as a comprehensive taxonomy intended to capture multiple aspects of PRP preparation and clinical application. The MARSPILL framework was proposed as a comprehensive taxonomy intended to capture multiple aspects of PRP preparation and clinical application.

Compared with PAW and DEPA, MARSPILL extends characterization beyond cellular composition by incorporating preparation and procedural variables. This broader scope improves descriptive completeness but also increases complexity, which may limit routine implementation [17].

### 3.4. MIBO Recommendations

Unlike PAW, DEPA and MARSPILL, the MIBO recommendations do not constitute a PRP classification system but rather a reporting standard. MIBO defines the minimum information that should be disclosed in studies involving orthobiologic products, including preparation methods, donor and recipient characteristics, processing conditions, characterization assays, routes of administration, and outcome measures.

Application of MIBO criteria to tendon-related studies has demonstrated persistent underreporting of preparation parameters and product characteristics, including baseline platelet counts, leukocyte characterization, centrifugation conditions, and the interval between preparation and administration [18,19].

Consequently, MIBO has emerged as a practical benchmark for assessing reporting quality and reproducibility in orthobiologic studies. The Q-PRP framework proposed in Section 10 should therefore be viewed as complementary to, rather than competitive with, the MIBO recommendations.

### 3.5. Strengths and Limitations of Current Systems

Collectively, these four frameworks address complementary but only partially overlapping aspects of PRP variability. PAW and DEPA primarily focus on cellular composition and platelet dose, whereas MARSPILL expands the analysis to preparation workflow and procedural variables. MIBO, in contrast, emphasizes reporting completeness and methodological transparency.

Despite their value, existing systems capture only partially overlapping dimensions of PRP heterogeneity and do not provide an integrated description of the administered product, its molecular characteristics, procedural context, and clinical application. Moreover, commercial preparation systems may generate substantially different cellular products despite being described within the same broad PRP category [8,9,10].

Classification systems therefore remain valuable for categorization and comparison but cannot substitute for comprehensive reporting of the biological product and its clinical context [6,8,18,19].

This persistent gap between classification and comprehensive biological characterization provides the rationale for the Q-PRP framework proposed in Section 10, which integrates quantitative descriptors with core and extended reporting requirements to improve reproducibility and cross-study comparability.

The main characteristics, advantages, and limitations of the currently available PRP classification and reporting systems are summarized in Table 1.

## 4. Cellular Composition of PRP

Although PRP is commonly defined by platelet enrichment, its biological activity reflects the combined influence of platelets, leukocytes, residual erythrocytes, and soluble mediators. Variation in these components contributes substantially to product heterogeneity and supports quantitative characterization of the administered preparation rather than reliance on platelet concentration alone [5,8,10].

### 4.1. Platelet Concentration Versus Total Platelet Dose

Early definitions of PRP emphasized platelet concentration, with approximately 1 × 10^6^ platelets/µL frequently cited as a therapeutic benchmark. However, concentration alone does not quantify the biological exposure delivered to the target tissue. Total platelet dose—the absolute number of platelets administered—also depends on the injected volume and therefore provides a more informative quantitative descriptor of platelet exposure. This concept was incorporated into the DEPA classification and has increasingly been emphasized in PRP standardization approaches [6,8,10,11].

Commercial PRP systems generate substantial differences in platelet recovery and total delivered dose, while higher platelet yields may also be accompanied by greater leukocyte and erythrocyte carry-over. Platelet dose should therefore be interpreted together with the cellular composition and purity of the final product [9,10].

The relationship between platelet dose and clinical efficacy remains incompletely defined and is unlikely to follow a simple linear dose–response pattern. Moreover, interindividual differences in baseline platelet counts may generate different final products even when identical preparation protocols are used. These observations support direct quantification of the administered product rather than inference from preparation method alone [5,11,12].

### 4.2. Leukocyte-Rich Versus Leukocyte-Poor PRP

Leukocyte content is one of the most debated determinants of PRP biology. LR-PRP and LP-PRP may generate different inflammatory environments, but the biological effect of leukocytes is strongly dependent on cell subtype, tissue context, and disease stage [6,13,14].

Leukocytes are active biological mediators rather than passive contaminants. Monocytes and macrophages participate in debris clearance and resolution of inflammation, whereas neutrophils can release reactive oxygen species, proteolytic enzymes, and pro-inflammatory mediators. Leukocyte composition may therefore support or impair repair depending on the balance among cell populations and the local biological environment [13,14,15,16].

Lymphocytes may further contribute to immunomodulation through cytokine-mediated interactions with resident and infiltrating cells; importantly, their relative abundance can vary substantially among PRP preparations, while their specific contribution to PRP-mediated tendon healing remains less well characterized than that of neutrophils and monocytes [20,21].

Clinical comparisons between LR-PRP and LP-PRP have not established consistent superiority of either formulation across tendon disorders, suggesting that total leukocyte content alone is insufficient to predict therapeutic response. Disease-specific clinical evidence is discussed in Section 8 [3,4,5].

The binary LR-PRP/LP-PRP distinction also treats leukocytes as a homogeneous population despite variable proportions of neutrophils, monocytes, and lymphocytes with distinct biological functions. Differential leukocyte characterization may therefore provide more meaningful information than total leukocyte content alone [6,13,14].

### 4.3. Red Blood Cell Contamination: The Overlooked Variable

Residual red blood cell (RBC) contamination is a potentially relevant but frequently overlooked determinant of PRP biology. Erythrocyte degradation can release free hemoglobin, iron, and reactive oxygen species, promoting oxidative stress, inflammatory signaling, and cellular toxicity. Experimental evidence therefore suggests that RBC contamination may adversely modify the local regenerative environment rather than acting as a biologically inert contaminant [5,8,10].

Higher platelet recovery may be accompanied by greater erythrocyte carry-over, creating a trade-off between platelet yield and product purity. Despite its potential biological relevance, RBC contamination remains inconsistently quantified in clinical studies, limiting comparison among PRP preparations [9,10].

RBC content is incorporated into existing characterization systems, including DEPA through its purity domain and MARSPILL through explicit erythrocyte reporting. However, no validated threshold currently defines clinically relevant RBC contamination [8,17].

### 4.4. Cellular Purity and Biologic Activity

Cellular purity provides an integrative descriptor of PRP composition but should not be interpreted simply as the absence of non-platelet cells. Platelet recovery, leukocyte composition, and erythrocyte contamination are interdependent characteristics, and maximizing platelet yield may increase unwanted cellular carry-over. Accordingly, purity must be interpreted together with platelet dose and differential cellular composition rather than as an isolated marker of product quality [8,9,10,17].

These considerations support quantitative reporting of the final PRP product rather than reliance on nominal product categories or preparation devices. Platelet dose, leukocyte subsets, and erythrocyte contamination provide complementary information that is necessary to interpret biological activity and compare clinical studies [5,8,10,18].

**Figure 1:**
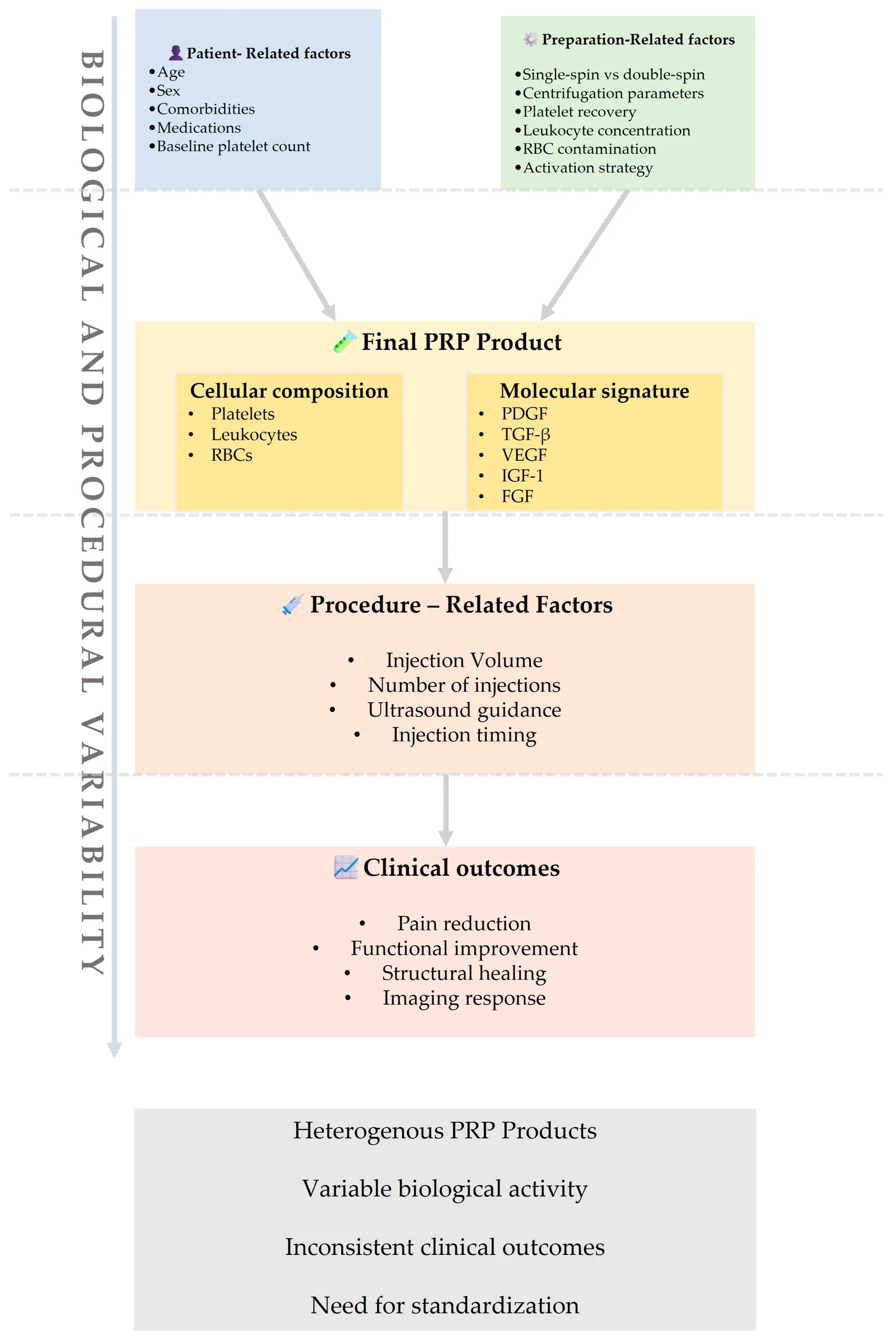
Major sources of variability influencing PRP composition and clinical outcomes in tendon healing. Patient-related, preparation-related, product-related, and procedure-related factors collectively determine the cellular composition and molecular profile of PRP. Variability at each stage contributes to heterogeneous biological activity and inconsistent clinical outcomes, highlighting the need for standardized PRP characterization.

The major cellular components of PRP and their respective biological contributions to tendon healing are summarized in Figure 1.

## 5. Growth Factor Profiles and Molecular Mediators

The regenerative activity of PRP is mediated by a complex platelet secretome containing growth factors, cytokines, chemokines, and other bioactive molecules. Among the principal growth factors implicated in tendon repair, PDGF, TGF-β1, VEGF, IGF-1, and bFGF regulate complementary processes including cell recruitment, extracellular matrix synthesis, angiogenesis, and tissue remodeling. Their biological effects depend on relative abundance, temporal release, and interaction with the local inflammatory environment rather than on the concentration of any single mediator [12,22,23,24].

### 5.1. Platelet α-Granules and Growth Factor Release

Following activation, platelets release a complex secretome from α-granules, dense granules and lysosomes. α-Granules represent the principal reservoir of regenerative proteins, including PDGF, TGF-β1, VEGF, IGF-1 and bFGF, whereas dense granules contain mediators such as ADP, ATP, calcium and serotonin that contribute to platelet activation and signaling. Together, these secretory compartments regulate inflammation, angiogenesis, cell proliferation and extracellular matrix remodeling [12,22,23].

Growth factor release is dynamic and depends partly on the mode of platelet activation. Endogenous tissue signals and exogenous activators can generate different degranulation and release kinetics, thereby modifying the temporal availability of regenerative mediators. Because activation strategies are examined separately in Section 7, the present section focuses on the principal growth factors relevant to tendon repair [12,23].

### 5.2. Platelet-Derived Growth Factor (PDGF)

PDGF is an early mediator of tendon repair and acts primarily as a chemoattractant and mitogen for tenocytes, fibroblasts, and progenitor cells. Among its isoforms, PDGF-BB has received particular attention because activation of PDGF receptors stimulates signaling pathways including PI3K/Akt and MAPK/ERK, promoting cell migration, proliferation, collagen synthesis, angiogenesis, and early extracellular matrix formation [22,23,24].

Experimental evidence further supports PDGF-mediated enhancement of tenocyte viability, migration, and collagen production, reinforcing its role in establishing the cellular and matrix environment required for subsequent tendon remodeling [22,23].

### 5.3. Transforming Growth Factor-β (TGF-β)

TGF-β, particularly TGF-β1, is a major regulator of extracellular matrix synthesis and tenogenic differentiation. Signaling predominantly through the canonical SMAD2/3 pathway, with contributions from non-canonical pathways, TGF-β promotes tenocyte proliferation, collagen and proteoglycan synthesis, progenitor-cell differentiation, and matrix deposition during tendon repair [22,23,24].

TGF-β1 activity is strongly time- and dose-dependent. While controlled signaling supports matrix restoration, persistent activation of the TGF-β/SMAD pathway can promote myofibroblast differentiation, excessive extracellular matrix deposition, and fibrotic remodeling. Its regenerative value therefore depends on appropriately regulated signaling rather than maximal exposure [22,23].

### 5.4. Vascular Endothelial Growth Factor (VEGF)

VEGF is the principal angiogenic mediator among the major PRP-associated growth factors. Released by activated platelets and local cells in response to injury and hypoxia, VEGF promotes endothelial proliferation, migration, and microvascular formation, thereby supporting oxygen and nutrient delivery during the proliferative phase of tendon repair [22,23,24].

VEGF also exhibits a context-dependent dual role. Transient angiogenesis supports regeneration, whereas persistent neovascularization is associated with chronic tendinopathy and may accompany sensory nerve ingrowth and pain. Effective repair therefore requires temporally controlled vascular remodeling rather than sustained angiogenic stimulation [22,23].

### 5.5. IGF-1 and bFGF: Anabolic and Remodeling Mediators

IGF-1 supports the anabolic and remodeling phases of tendon repair by promoting tenocyte proliferation, cellular survival, collagen synthesis, and matrix organization. These effects are mediated predominantly through PI3K/Akt and mTOR signaling and contribute to restoration of tendon structure and mechanical properties [22,23,24].

bFGF promotes proliferation and migration of fibroblasts, tenocytes, and tendon-derived progenitor cells and contributes to extracellular matrix remodeling. Controlled bFGF signaling can support regeneration, whereas excessive or prolonged stimulation may favor disorganized tissue repair [22,23].

Together, IGF-1 and bFGF sustain cellular and matrix remodeling during later stages of tendon repair, complementing the earlier recruitment, matrix-synthesis, and angiogenic effects mediated predominantly by PDGF, TGF-β and VEGF [22,23,24].

The principal growth factors contained in PRP, their biological sources, functions, and potential clinical relevance during tendon healing are summarized in Table 2.

### 5.6. Integrated Growth Factor Profiles During Tendon Healing

PRP-derived growth factors act as an integrated and temporally regulated signaling network rather than as isolated mediators. Accordingly, the molecular profile of a preparation reflects not only the abundance of individual growth factors but also their relative proportions, release kinetics, and interactions. Preparations with similar platelet concentrations may therefore exhibit different biological activity because of differences in cellular composition, preparation, and activation [12,22,23,24].

**Figure 2:**
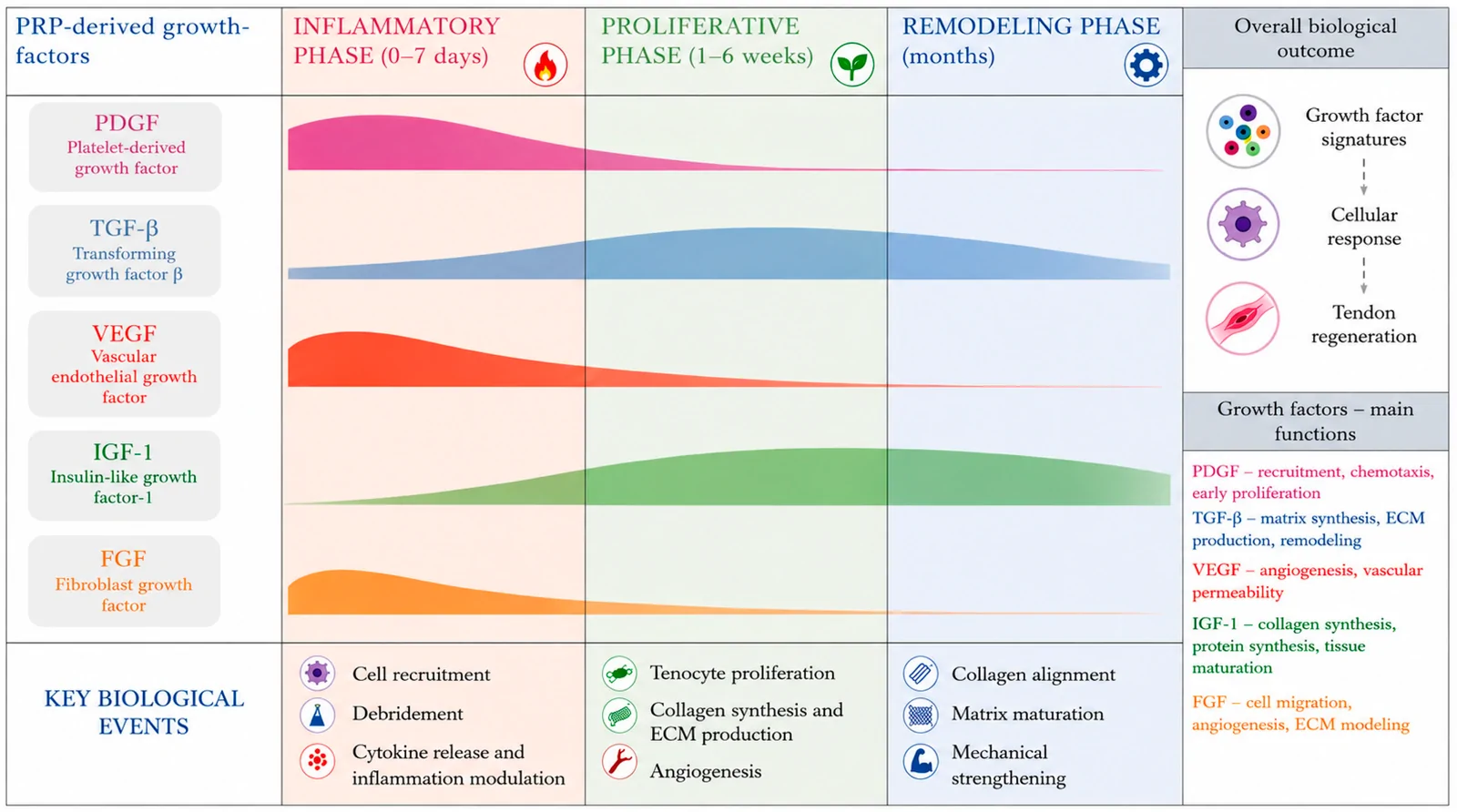
Coordinated actions of the principal PRP-derived growth factors during the different phases of tendon healing.

Temporal coordination is particularly relevant during tendon healing: PDGF predominates in early cell recruitment, TGF-β1 supports matrix synthesis and differentiation, VEGF promotes vascular adaptation, and IGF-1 and bFGF contribute to matrix maturation and remodeling. These activities overlap substantially rather than occurring as discrete sequential events (Table 3 and Figure 2) [22,23,24].

Because molecular signaling is further influenced by the inflammatory microenvironment, cellular composition, and activation strategy, growth factor concentrations alone cannot fully define PRP activity. Molecular profiling may provide additional mechanistic information, but its current analytical complexity limits routine clinical implementation; accordingly, it is best considered an extended rather than mandatory component of PRP characterization [12,23,24].

The activity of these anabolic mediators is closely influenced by the inflammatory environment, providing the rationale for examining cytokine balance in the following section [12,15,16].

## 6. Cytokines and the Inflammatory Balance of PRP

PRP acts not only as a source of anabolic growth factors but also as an immunomodulatory biologic. Cytokines released by platelets, leukocytes, and resident tendon cells regulate the initiation, magnitude, and resolution of inflammation. Controlled inflammatory activation supports debris clearance and recruitment of reparative cells, whereas persistent signaling promotes extracellular matrix degradation and chronic tendon degeneration [15,16,26,27,28].

The cytokine profile of PRP varies with cellular composition, donor characteristics, preparation, and activation. In particular, leukocyte content influences the balance between pro-inflammatory mediators such as IL-1β, IL-6, and TNF-α and pro-resolving signals such as IL-10, making inflammatory balance more informative than the concentration of any single cytokine [26,29].

### 6.1. Interleukin-1β (IL-1β)

IL-1β is a major early pro-inflammatory mediator released predominantly by activated macrophages and neutrophils following tendon injury. Although transient signaling contributes to the initiation of repair, persistent IL-1β activity promotes a catabolic tenocyte phenotype characterized by increased IL-6 and MMP expression, reduced type I collagen synthesis, and progressive extracellular matrix degradation [15,16,26,28].

IL-1β interacts with TNF-α and downstream cytokine pathways to amplify local inflammation and may counteract anabolic signaling when activation becomes excessive or prolonged. Its biological effect is therefore dependent on timing and the surrounding cytokine milieu rather than on its isolated presence [16,26,28,29].

### 6.2. Interleukin-6 (IL-6)

IL-6 is a pleiotropic cytokine with both inflammatory and regenerative functions in tendon healing. Following injury, it is produced by inflammatory cells, tenocytes, and endothelial cells and contributes to leukocyte recruitment, extracellular matrix turnover, cell proliferation, and the transition from inflammation toward tissue repair [15,16,26,27,28].

The context-dependent effects of IL-6 are partly related to signaling mode: classical signaling is generally associated with homeostatic and regenerative responses, whereas trans-signaling may sustain inflammation. Thus, elevated IL-6 within a PRP preparation should not be interpreted in isolation but in relation to the broader inflammatory environment and stage of tendon healing [15,16,26,29].

### 6.3. Tumor Necrosis Factor-α (TNF-α)

TNF-α is an upstream inflammatory mediator rapidly induced after tendon injury. Transient signaling contributes to initiation of repair, whereas sustained exposure promotes a catabolic tenocyte phenotype characterized by increased inflammatory mediators and matrix metalloproteinases, reduced type I collagen synthesis, MMP, and, under prolonged conditions, tendon-cell apoptosis [15,16,26,27].

TNF-α acts synergistically with IL-1β and promotes IL-6 expression and nuclear factor kappa B (NF-κB) activation, generating positive feedback that may sustain chronic inflammation. As with other inflammatory mediators, its biological relevance depends on the magnitude and duration of signaling rather than its presence alone [15,16,26,29].

### 6.4. Interleukin-10 (IL-10) and Inflammation Resolution

IL-10 is a principal endogenous mediator of inflammation resolution. Produced by regulatory immune-cell populations, it suppresses IL-1β, TNF-α, and NF-κB signaling, reduces matrix metalloproteinase expression, and supports macrophage transition toward pro-resolving phenotypes. These actions facilitate restoration of extracellular matrix homeostasis and progression from inflammation toward tissue remodeling [15,16,26,28].

The role of IL-10 illustrates that successful tendon repair requires active resolution rather than simple suppression of inflammation. PRP activity should therefore be interpreted according to the balance between inflammatory and pro-resolving signaling rather than the concentration of individual cytokines [16,29].

### 6.5. Biological Implications of LR-PRP Versus LP-PRP

The inflammatory profiles described above provide the biological basis for differences between LR-PRP and LP-PRP. Leukocyte-rich preparations generally contain greater numbers of inflammatory cells and higher concentrations of cytokines such as IL-1β, IL-6, and TNF-α, whereas leukocyte-poor preparations reduce this inflammatory component while retaining platelet-derived anabolic mediators. These formulations should therefore be regarded as biologically distinct products rather than stronger or weaker versions of the same intervention [16,26,29].

Neither formulation can be considered universally superior. The biological effect of leukocytes depends on their differential composition, disease context, and timing of treatment, while clinical response is additionally influenced by platelet dose, molecular profile, and procedural factors. This complexity supports characterization of leukocyte subsets rather than reliance on the binary LR-PRP/LP-PRP distinction alone [4,5,12,26,28].

**Figure 3:**
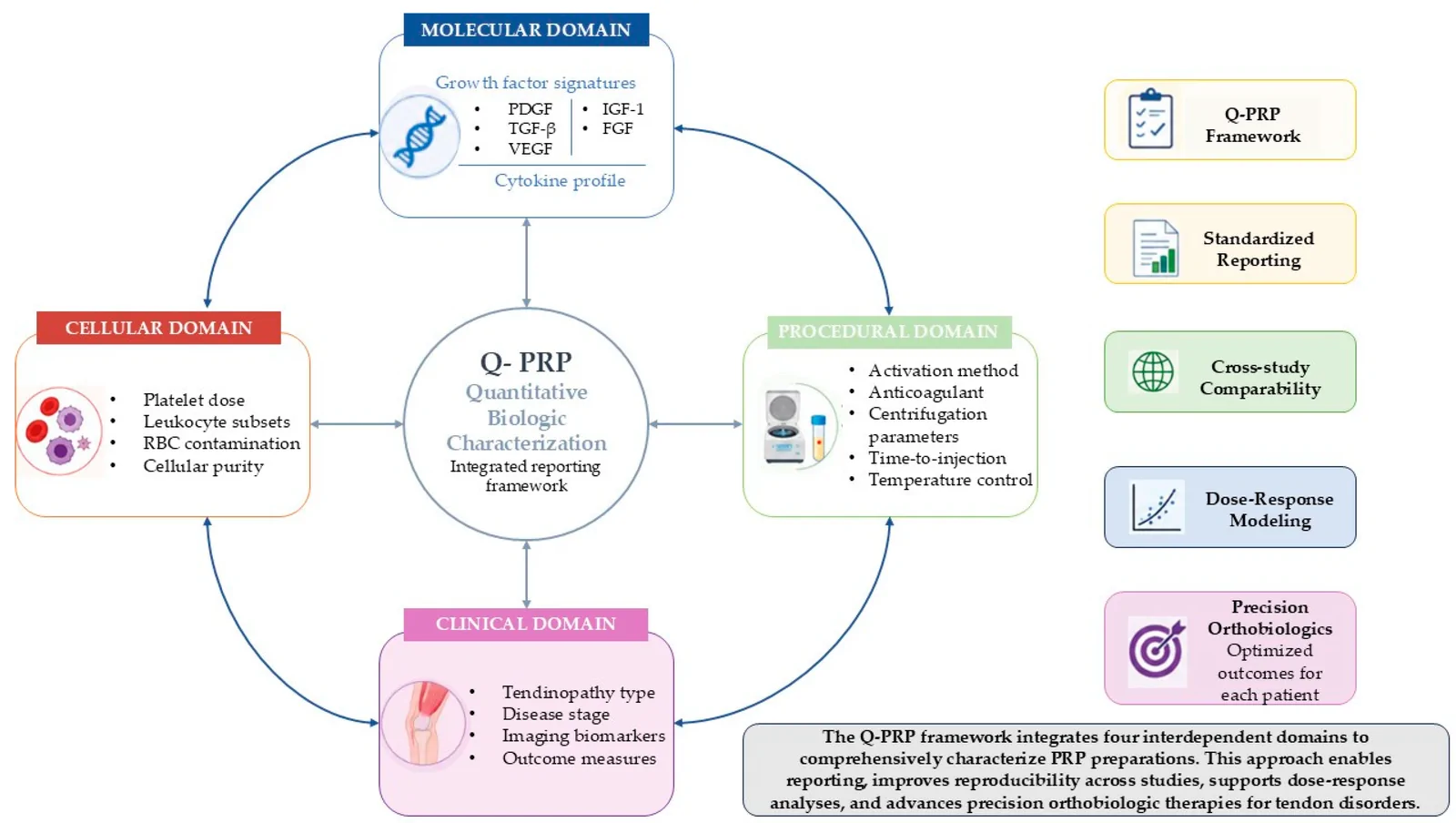
Cytokine balance as a determinant of the biological activity of PRP during tendon healing. PRP modulates both pro-inflammatory and anti-inflammatory cytokine networks. While IL-1β, TNF-α, and IL-6 contribute to inflammatory activation and matrix degradation when excessive or prolonged, IL-10 promotes inflammation resolution, macrophage polarization, and tissue repair. Functional tendon regeneration depends on maintaining an appropriate balance between these opposing biological responses.

Figure 3 illustrates the dynamic balance between pro-inflammatory and anti-inflammatory cytokines released or modulated by PRP and their contribution to the transition from inflammation toward functional tendon regeneration.

## 7. PRP Activation Strategies

Platelet activation is a major determinant of PRP biological activity because it initiates platelet degranulation and release of growth factors, cytokines, chemokines, and adhesive proteins. The activation method influences both the magnitude and kinetics of mediator release and may therefore modify the regenerative microenvironment established after PRP administration [30,31,32,33].

Activation may occur endogenously after PRP administration or be induced ex vivo using agents such as calcium chloride or thrombin. Because these approaches differ in degranulation kinetics, fibrin formation, and temporal mediator availability, activation strategy should be reported as an integral component of PRP characterization [30,32,34,35].

### 7.1. Endogenous Activation

Endogenous activation occurs after non-activated PRP is delivered to the target tissue, where platelets encounter collagen, locally generated thrombin, and other components of the injured extracellular matrix. This approach minimizes premature degranulation and allows mediator release to occur within the tissue microenvironment, potentially producing more gradual and physiologically synchronized release kinetics [30,31,33]. This dependency represents a potential limitation in chronic tendinopathy, where the local biological and extracellular matrix environment differs substantially from that of an acute tendon injury. Because endogenous platelet activation depends on local platelet agonists such as exposed collagen and thrombin, together with an intact coagulation environment, insufficient or heterogeneous availability of these signals may result in less predictable activation and growth-factor release after administration of non-activated PRP [36,37].

Endogenous activation avoids additional activating agents but introduces tissue-dependent variability because the onset and magnitude of platelet activation depend on local biological conditions. Current evidence does not establish its superiority over exogenous activation, and its principal distinction lies in preserving tissue-dependent activation and release kinetics [30,33,34].

### 7.2. Calcium Chloride Activation

Calcium chloride (CaCl_2_) induces platelet activation indirectly by restoring calcium-dependent coagulation after citrate anticoagulation, thereby promoting endogenous thrombin generation, platelet degranulation, and fibrin polymerization. Compared with direct thrombin activation, CaCl_2_ generally produces more gradual mediator release while generating a fibrin scaffold that may retain growth factors and support cell migration and extracellular matrix deposition [30,32,33].

CaCl_2_ is inexpensive and avoids exposure to exogenous biological proteins, but activation kinetics vary with calcium concentration, anticoagulant, and preparation protocol. Although its progressive release profile is biologically attractive, clinical superiority over alternative activation strategies has not been established [32,33,34].

### 7.3. Thrombin Activation

Thrombin induces rapid platelet degranulation and fibrin polymerization, resulting in an early burst of platelet-derived mediators and formation of a dense fibrin matrix. Compared with endogenous or calcium-mediated activation, its principal biological distinction is the rapid availability of growth factors and cytokines [30,31,32,35].

Historically, bovine thrombin was used because of its rapid and reproducible effects, but concerns regarding immunogenicity have favored autologous thrombin or alternative activators. These concerns are particularly relevant to bovine thrombin, exposure to which can induce antibodies against bovine thrombin and contaminating coagulation proteins, particularly factor V/Va, with potential cross-reactivity against human coagulation factors and consequent hemostatic abnormalities. Autologous thrombin avoids xenogeneic antigen exposure and therefore represents a biologically preferable alternative when thrombin-mediated activation is required [38,39]. Although thrombin provides controlled and rapid activation, an early burst of mediator release is not necessarily advantageous for the prolonged biological processes involved in tendon repair, and no consistent superiority has been established [31,32,33,34].

### 7.4. Should Platelets Be Activated Before PRP Administration?

Current evidence does not establish universal superiority of activated or non-activated PRP. Exogenous activation offers greater control over platelet degranulation and immediate mediator availability, whereas endogenous activation preserves tissue-dependent signaling and may provide more physiologically regulated release kinetics [30,31,32,33,34].

The choice of activation strategy should therefore reflect the intended biological response, target tissue, and clinical context rather than the assumption that one approach is intrinsically superior. Importantly, activation should be interpreted as one component of PRP formulation and reported alongside cellular composition and other preparation variables [33,34,35].

### 7.5. Activation Methods as Determinants of Growth Factor Release Kinetics

The principal biological distinction among activation strategies lies in their release kinetics and resulting fibrin architecture. Thrombin produces rapid degranulation and early mediator availability, CaCl_2_ induces more progressive release through endogenous thrombin generation, and endogenous activation delays release until platelets encounter the injured tissue. These differences may influence growth factor retention, diffusion, cellular recruitment, and extracellular matrix remodeling [30,31,32,33].

Accordingly, PRP preparations with similar platelet concentrations and cellular composition may exhibit different biological behavior depending on activation strategy. Standardized reporting should therefore specify whether activation was performed and, when applicable, the activating agent and protocol used [33,34,35].

**Figure 4:**
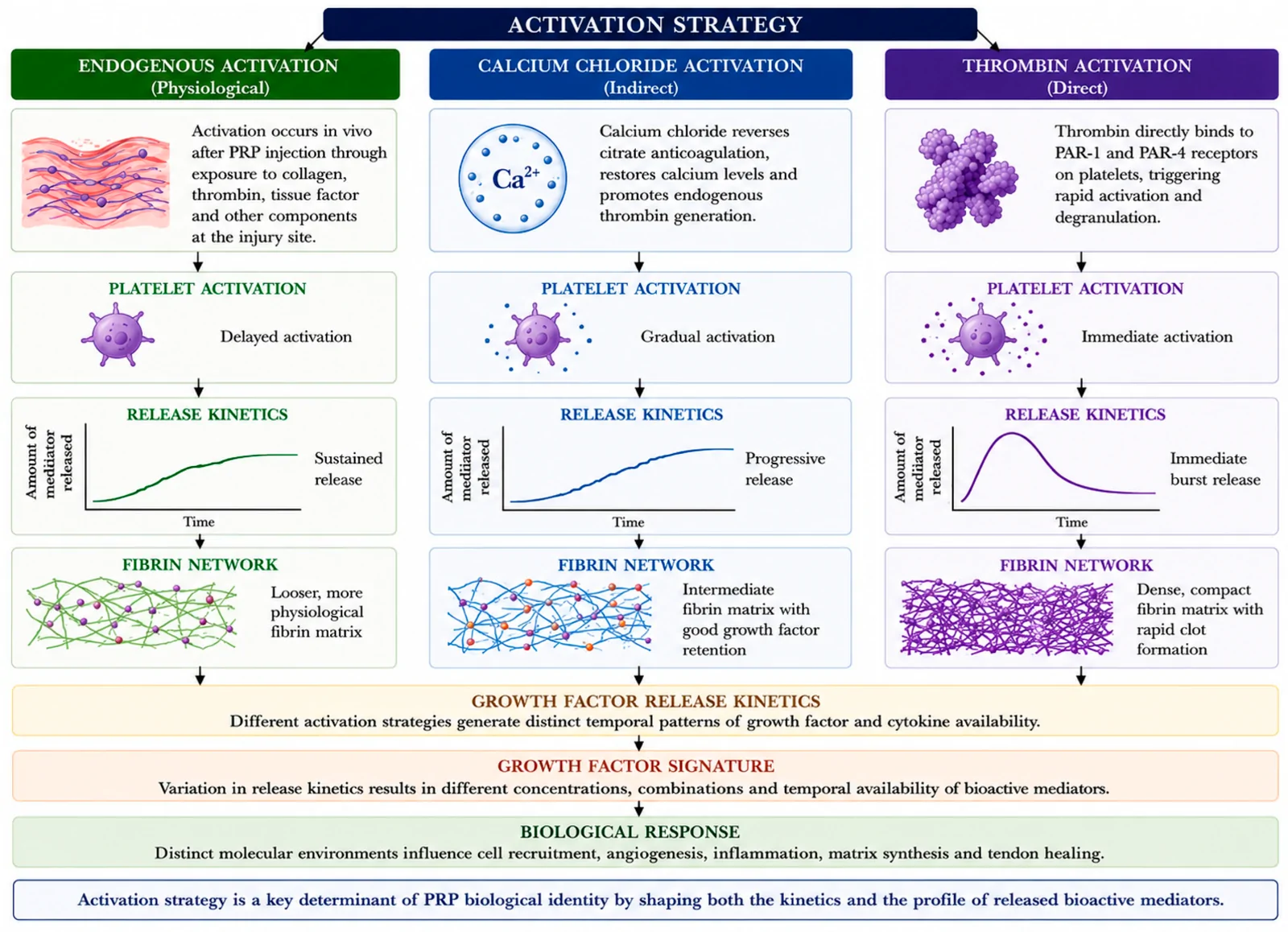
Influence of platelet activation strategies on platelet activation, growth factor release kinetics, and the biological response of PRP. Endogenous activation, calcium chloride activation, and thrombin activation produce distinct patterns of platelet degranulation, fibrin network formation, and temporal release of growth factors and cytokines. These activation-dependent differences generate unique molecular environments that influence growth factor availability, angiogenesis, inflammation, matrix synthesis, and tendon healing. Consequently, platelet activation strategy represents a key determinant of the biological identity and therapeutic potential of PRP.

The biological consequences of different platelet activation strategies on growth factor release kinetics and tendon healing are summarized in Figure 4.

## 8. Clinical Evidence for PRP in Major Tendon Disorders

Clinical outcomes after PRP therapy vary substantially among tendon disorders and across studies, reflecting differences in disease biology, PRP formulation, treatment protocols and methodological design. The following sections summarize the evidence for Achilles tendinopathy, patellar tendinopathy, rotator cuff disease and lateral epicondylitis, with emphasis on disease-specific efficacy and the principal factors limiting interpretation of current evidence [40,41]. Where available, pooled quantitative estimates from meta-analyses are provided to contextualize the magnitude and certainty of reported clinical effects.

### 8.1. Achilles Tendinopathy

Despite a plausible regenerative rationale, clinical evidence for PRP in chronic Achilles tendinopathy remains inconsistent. Randomized trials and pooled analyses have generally failed to demonstrate clinically meaningful superiority over placebo or established conservative treatment for pain or functional outcomes. In a recent meta-analysis including 422 patients with chronic midportion Achilles tendinopathy, PRP produced no significant improvement in VISA-A scores compared with control at 3, 6, or 12 months (*p* = 0.29, 0.42, and 0.57, respectively) [42].

Interpretation is limited by heterogeneity in disease chronicity, PRP formulation, injection protocols, and rehabilitation, while advanced structural degeneration may further restrict biological responsiveness. Current evidence therefore does not support routine PRP use as a first-line treatment for chronic Achilles tendinopathy, although better patient stratification and standardized PRP characterization may help identify responsive subgroups [40,41,43,44,45].

### 8.2. Patellar Tendinopathy

Evidence for PRP in patellar tendinopathy is generally more favorable than for Achilles tendinopathy, with several clinical studies reporting improvements in pain and function, particularly in patients with chronic symptoms refractory to conservative treatment. However, treatment effects remain variable, and the overall certainty of evidence is moderate rather than definitive [40,41,43,44,45].

Substantial heterogeneity remains in PRP composition, injection number, and rehabilitation protocols, and current evidence does not establish superiority of a specific leukocyte formulation. PRP may therefore represent a therapeutic option for selected patients with chronic patellar tendinopathy, particularly when integrated with structured loading-based rehabilitation, but standardized trials are still required to define the optimal treatment protocol [40,41,43,44,45,46].

### 8.3. Rotator Cuff Tendinopathy and Surgical Repair

The clinical role of PRP in rotator cuff disease differs according to treatment setting. For non-operative rotator cuff tendinopathy, available trials show inconsistent improvements in pain and function and do not establish reliable superiority over conventional treatments; consequently, routine PRP injection cannot currently be recommended as first-line therapy [40,41,44,45].

Evidence is more favorable for PRP used as an adjunct to arthroscopic rotator cuff repair. In a meta-analysis of randomized controlled trials, intraoperative PRP application at the tendon–bone interface significantly reduced the postoperative re-tear rate compared with control (RR 0.38, 95% CI 0.22–0.68; *p* = 0.0009), although improvements in functional outcomes were more modest [43]. Interpretation remains limited by substantial variation in PRP formulation, activation, application site, and surgical technique [43,45,47].

Thus, PRP appears more promising as a biological adjunct to surgical repair than as an isolated treatment for chronic rotator cuff tendinopathy. Future studies should determine which formulations, delivery strategies, and patient populations are associated with reproducible structural and clinical benefit [41,45,46,48].

### 8.4. Lateral Epicondylitis

Lateral epicondylitis is among the most extensively studied tendon disorders in the PRP literature. Randomized trials and meta-analyses suggest that PRP may provide greater medium- and long-term improvement in pain and function than corticosteroid injection, whereas corticosteroids may provide greater short-term symptom relief; however, heterogeneity across studies prevents definitive conclusions regarding the optimal PRP formulation [40,41,44,45].

The potential longer-term benefit of PRP is compatible with its regenerative rather than primarily anti-inflammatory mechanism, but considerable variability in platelet dose, leukocyte composition, injection technique, and rehabilitation limits comparison among trials. Importantly, available evidence does not establish superiority of LR-PRP over LP-PRP in lateral epicondylitis, and leukocyte content should not be interpreted independently of other product and treatment characteristics [40,43,45,46,48].

Overall, PRP can be considered a therapeutic option for selected patients with chronic lateral epicondylitis, particularly after failure of conservative treatment, but further standardized studies are required to determine the formulation and treatment protocol associated with the greatest clinical benefit [41,44].

### 8.5. Understanding the Sources of Clinical Heterogeneity

Across tendon disorders, clinical heterogeneity reflects the interaction of product-related, patient-related, and treatment-related variables. Differences in platelet dose, leukocyte composition, activation, disease chronicity, injection technique, rehabilitation, and outcome assessment limit direct comparison among studies and may obscure formulation-specific treatment effects [40,41,43,44,45,46]. Importantly, disease-specific comparisons according to leukocyte composition or activation strategy remain limited because many clinical trials incompletely characterize the administered PRP product; consequently, current evidence does not permit reliable formulation-specific efficacy estimates across most tendon disorders.

A major limitation remains the incomplete biological characterization of PRP used in clinical trials, which frequently results in biologically distinct preparations being evaluated under the same generic designation. Improving clinical interpretation therefore requires standardized reporting that links the administered product to the patient, disease, and treatment context—a central objective of the Q-PRP framework proposed in Section 10 [5,12,45,46,48].

## 9. Imaging Biomarkers of PRP Response

Clinical outcomes do not necessarily reflect the structural and biological changes occurring within a healing tendon. Imaging therefore provides a complementary approach for assessing tissue remodeling after PRP therapy, with quantitative ultrasound and magnetic resonance imaging (MRI)-based techniques offering potential objective biomarkers for treatment monitoring and research standardization [41,49].

### 9.1. Conventional Ultrasound

Conventional ultrasound provides an accessible method for serial assessment of tendon thickness, echogenicity, fibrillar architecture, and focal structural abnormalities and remains particularly useful for image-guided PRP administration and longitudinal monitoring [49].

Its principal limitation as an outcome measure is the imperfect relationship between structural appearance and clinical recovery: persistent sonographic abnormalities may coexist with symptomatic improvement, while symptoms may occur with limited morphological change. Conventional ultrasound should therefore complement rather than substitute clinical outcome assessment [41,49].

### 9.2. Doppler Ultrasound and Elastography

Doppler ultrasound and elastography extend conventional imaging by assessing vascular and mechanical characteristics of tendon tissue. Doppler techniques can quantify intratendinous neovascularization, although increased vascularity may reflect either chronic tendinopathy or transient reparative activity; interpretation therefore depends on timing and clinical context [41,49].

Elastography provides quantitative assessment of tendon mechanical properties and may detect changes in stiffness associated with collagen remodeling and tissue maturation. However, Doppler and elastographic measurements remain sensitive to acquisition technique, loading conditions, and equipment, while validated normative values and clinically meaningful thresholds are still lacking. Standardized acquisition protocols are therefore required before these measures can serve as reproducible PRP-response biomarkers [41,49].

### 9.3. MRI and Quantitative MRI

MRI provides detailed assessment of tendon morphology and is particularly useful for deep tendons and complex anatomical regions. It can document tendon thickness, signal abnormalities, structural defects, and surrounding tissue changes during follow-up; however, as with ultrasound, structural MRI findings correlate imperfectly with pain and functional recovery [41,49].

Quantitative MRI techniques, including T2 mapping and ultrashort echo time imaging, may provide more objective information on collagen organization, water content, and matrix integrity than conventional morphological assessment. These approaches remain predominantly research tools but represent promising candidates for standardized evaluation of biological tendon remodeling after PRP therapy [41,49].

### 9.4. Emerging Imaging Biomarkers: Radiomics and Artificial Intelligence (AI)

Radiomics enables extraction of quantitative features describing image texture, signal heterogeneity, shape, and spatial characteristics that may capture tissue changes beyond visual assessment. Combined with AI and machine-learning approaches, such imaging features could eventually be integrated with clinical and PRP characteristics to investigate treatment-response phenotypes and predictive models [49].

These approaches remain investigational and require standardized acquisition, reproducible feature extraction, and external validation before clinical implementation. Their current relevance to PRP research is therefore primarily as potential research tools for quantitative and individualized assessment rather than established clinical biomarkers [49].

### 9.5. Imaging Biomarkers as Objective Outcome Measures

Imaging biomarkers should complement rather than replace patient-reported and functional outcomes because clinical improvement and structural regeneration represent related but non-equivalent dimensions of treatment response. Combining morphological, vascular, mechanical, and compositional imaging measures may therefore provide a more complete assessment of tendon recovery [41,49].

For PRP research, the principal value of quantitative imaging lies in linking objective tissue response to a defined biological product. Standardized imaging protocols could enable correlations between tendon remodeling and PRP characteristics such as platelet dose, leukocyte composition, and activation strategy, thereby complementing clinical endpoints and strengthening biological interpretation of treatment response [41,49].

## 10. Toward Standardized and Quantifiable PRP Characterization: The Proposed Q-PRP Framework

### 10.1. Why Current PRP Reporting Remains Insufficient

Despite extensive clinical investigation, interpretation of PRP studies remains limited by incomplete and inconsistent characterization of the administered product. Existing systems such as PAW, DEPA, and MARSPILL, together with MIBO reporting recommendations, have improved standardization but capture only partially overlapping cellular, procedural, and reporting domains. Essential variables—including platelet dose, leukocyte differential, erythrocyte contamination, activation, injected volume, and rehabilitation—remain inconsistently reported, limiting reproducibility and cross-study comparison [5,6,8,9,10,17,18,19].

These limitations support a reporting approach that integrates product characteristics with procedural and clinical context. We therefore propose the Q-PRP framework as an operational reporting model designed to improve biological characterization, reproducibility, and interpretation of PRP studies without replacing existing classification systems or reporting recommendations.

### 10.2. Essential Variables for Biologically Meaningful PRP Characterization

Q-PRP organizes biologically meaningful PRP characterization into four complementary domains: cellular, molecular, procedural, and clinical variables. These domains describe both the administered biological product and the context required to interpret its effects.

Cellular Variables

Cellular variables include platelet concentration and absolute platelet dose, leukocyte composition, and residual erythrocyte contamination. Together, these parameters provide a more informative description of the administered product than platelet enrichment or the LR-PRP/LP-PRP designation alone [6,8,9,10,13,14].

Molecular Variables

Molecular variables include growth-factor and cytokine profiles that may provide mechanistic information beyond cellular composition. Because comprehensive molecular profiling is not routinely feasible, these variables are incorporated primarily within the extended Q-PRP dataset rather than the minimum clinical reporting standard [12,22,23,24].

Procedural Variables

Procedural variables include preparation method, anticoagulant, activation strategy, injected volume, number of injections, and image guidance, all of which may influence reproducibility and biological exposure [30,31,32,33,34,35].

Clinical Variables

Clinical variables describe the treatment context, including tendon disorder, disease chronicity, rehabilitation, follow-up, and outcome assessment. These variables are necessary to distinguish product-related effects from differences in patient selection and treatment protocols [40,41,43,44,45,46,47,48].

To operationalize these domains, Q-PRP uses a two-level protocol comprising a core dataset representing the minimum reporting standard for clinical tendon-PRP studies and an extended dataset for advanced biological, molecular, imaging, or precision-medicine investigations. Core variables prioritize biological relevance, routine feasibility, and reproducibility, whereas extended variables provide additional mechanistic information. The proposed protocol is summarized in Table 4.

### 10.3. The Proposed Q-PRP Framework

Q-PRP is intended as an operational reporting framework rather than a new PRP classification system. It complements PAW, DEPA, MARSPILL and MIBO by specifying a minimum core dataset while allowing extended molecular and imaging characterization when appropriate. This two-level structure is designed to balance biological completeness with feasibility in routine clinical research.

The framework deliberately separates characterization of the PRP product from characterization of its clinical context while requiring both for meaningful interpretation. Cellular and procedural variables define the administered intervention, whereas patient, disease, rehabilitation and outcome variables describe the environment in which that intervention is evaluated. Extended molecular and imaging variables may further support mechanistic or precision-medicine analyses.

Host genetic variability is emerging as a potential modifier of PRP treatment response. Prospective studies in lateral elbow tendinopathy have reported associations between clinical response to PRP and single-nucleotide polymorphisms involving VEGFA and TGFβ1, as well as genes encoding PDGF receptors (PDGFRA and PDGFRB). Some PDGFRA variants have additionally been associated with platelet parameters in whole blood and PRP, suggesting that host genotype may influence both the administered product and the biological response to treatment. These findings remain preliminary and require independent validation before genetic profiling can be incorporated into routine clinical decision-making; however, genomic variables may represent a future component of extended Q-PRP characterization and precision-oriented PRP research [52,53,54,55].

By standardizing what is reported rather than introducing another categorical nomenclature, Q-PRP is intended to facilitate cross-study comparison, evidence synthesis, and investigation of dose–response and formulation–outcome relationships. Several limitations of the proposed Q-PRP framework should be acknowledged. The framework is evidence-informed but has not yet undergone prospective validation, and the relative clinical importance of individual variables remains uncertain. Implementation may also be constrained by laboratory availability, cost, and differences in local processing capabilities, particularly for extended molecular, genetic, and imaging variables. Accordingly, Q-PRP should currently be considered a standardized reporting framework rather than a validated scoring system or treatment-selection algorithm. Its value will ultimately depend on prospective application and external validation across independent clinical cohorts.

**Figure 5:**
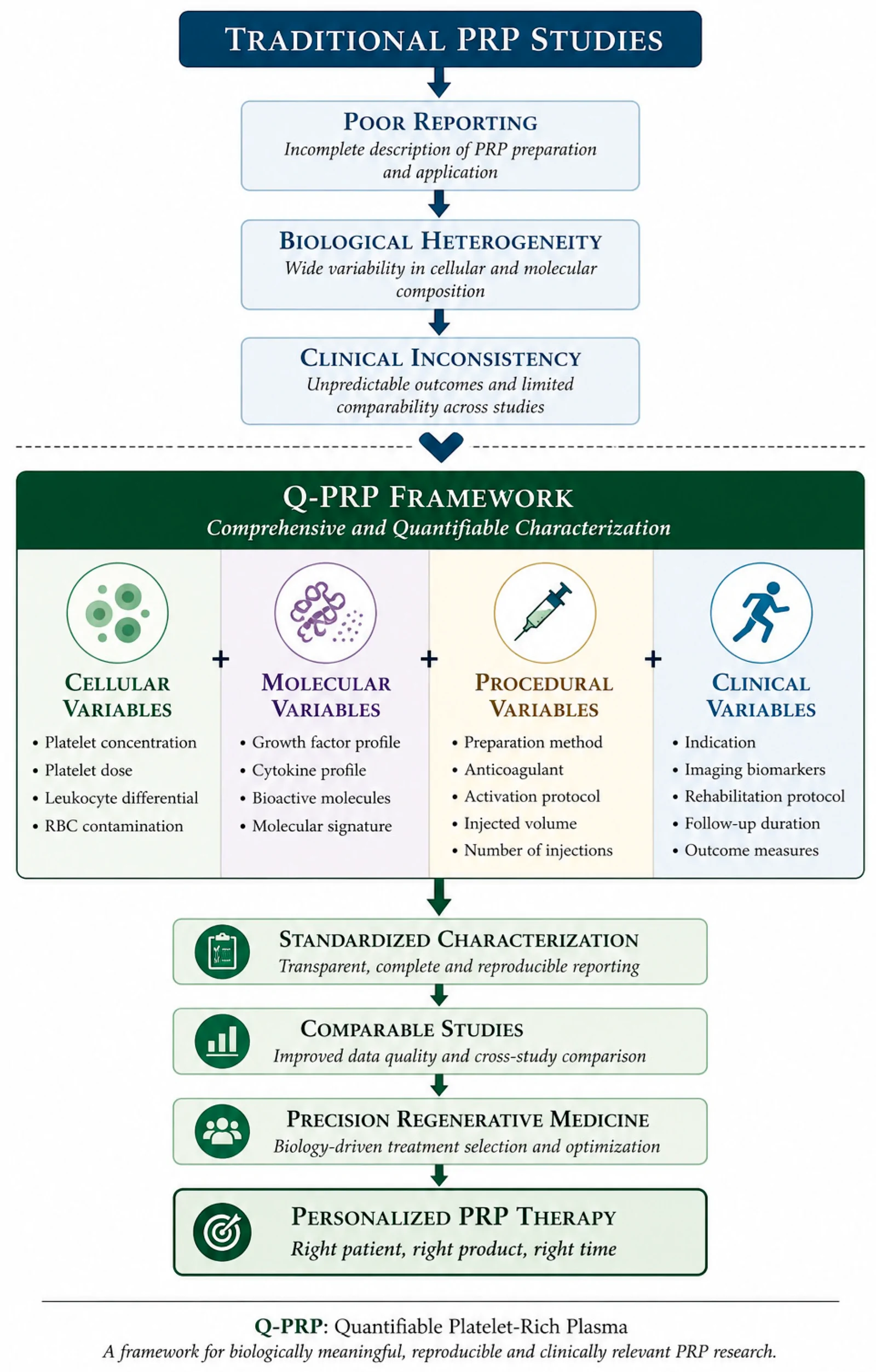
Proposed quantifiable platelet-rich plasma (Q-PRP) framework for standardized characterization and reporting of PRP in tendon studies. The framework integrates cellular, molecular, procedural, and clinical domains within a two-level reporting structure comprising core/minimum and extended/research variables.

The structure and principal domains of the proposed Q-PRP framework are illustrated in Figure 5.

### 10.4. Practical Recommendations for Future PRP Research

Future PRP research should prioritize standardized characterization and reporting rather than evaluating incompletely defined preparations in progressively larger trials. Based on the evidence reviewed here, four priorities emerge.

First, studies should report the Q-PRP core dataset, including quantitative cellular characterization and the essential preparation and administration variables required to reproduce the intervention. Extended molecular characterization should be incorporated when mechanistically relevant and technically feasible [5,6,8,9,10,17,18,19].

Second, treatment context should be standardized or explicitly documented, particularly injection protocol and post-treatment rehabilitation, because these factors may substantially modify tendon response independently of PRP composition [40,41,43,44,45,46,47,48].

Third, clinical trials should combine validated patient-reported and functional outcomes with objective imaging measures when appropriate. Quantitative imaging may strengthen assessment of tissue remodeling, but emerging techniques such as radiomics should remain research endpoints until adequately validated [41,49].

Finally, future studies should evaluate biologically characterized PRP formulations within defined tendon disorders and patient populations rather than treating PRP as a uniform intervention. Such designs are necessary to determine whether specific product characteristics are associated with reproducible treatment response [5,12,40,45].

Prospective application of standardized reporting frameworks will be necessary to determine whether improved biological characterization translates into greater reproducibility and clinically meaningful treatment stratification. Q-PRP should therefore be regarded as a proposed reporting framework requiring prospective evaluation and refinement rather than as a validated classification or treatment-selection tool.

## 11. Conclusions

PRP is a biologically complex and heterogeneous orthobiologic rather than a uniform therapeutic product. Its effects on tendon repair reflect interactions among platelet dose, leukocyte and erythrocyte content, molecular mediators, activation strategy, and the local tissue environment. Although experimental evidence provides a strong biological rationale for PRP, inconsistent product characterization and substantial variability in treatment protocols continue to limit reproducibility and interpretation of clinical outcomes.

Clinical efficacy appears to be indication-dependent, with more favorable evidence in selected conditions such as lateral epicondylitis and patellar tendinopathy, less consistent results in Achilles and non-operative rotator cuff tendinopathy, and potential benefit as an adjunct to rotator cuff repair. Importantly, current evidence does not support a universally superior PRP formulation, emphasizing the need to interpret treatment response in relation to both product characteristics and disease-specific context.

To address these limitations, we propose the Q-PRP framework as an operational reporting model that complements existing classification and reporting systems by integrating cellular, molecular, procedural, and clinical variables within core/minimum and extended/research datasets. Q-PRP is intended to improve reproducibility and cross-study comparison rather than to define treatment efficacy or replace established classifications, and it requires prospective application and external validation. Ultimately, progress in PRP research will depend on moving from the generic question of whether “PRP works” toward determining which biologically characterized formulation may be most appropriate for a defined tendon disorder and clinical context.

## Figures and Tables

**Table 1:** Current classification and reporting frameworks for platelet-rich plasma.

Classification/Reporting Framework	Main Variables Evaluated	Key Strengths	Major Limitations
PAW classification [6,8].	Absolute platelet number/platelet concentration; platelet activation; white blood cell content, including neutrophil consideration	Simple, clinically intuitive, and useful for comparing PRP formulations based on three core variables	Does not include total injected platelet dose, RBC contamination, growth factor profile, or delivery protocol
DEPA classification [6,8].	Dose of injected platelets; efficiency of production; purity of PRP; activation process	Introduces total platelet dose and product purity, addressing limitations of concentration-only descriptions	Does not fully integrate leukocyte subsets, growth factor profile, clinical indication, imaging guidance, or injection protocol
MARSPILL classification [6,8].	Method; activation; red blood cells; spin; platelets; image guidance; leukocytes; light activation	Broad framework including preparation method, cellular composition, and delivery guidance	Relatively complex; limited routine adoption; does not directly link molecular profile with clinical outcomes
MIBO guidelines [6,8].	Minimum reporting items for PRP studies, including study design, recipient details, injury characteristics, preparation, activation, delivery, and outcomes	Improves transparency, reproducibility, and critical appraisal of PRP studies	Reporting guideline rather than a PRP classification system; adherence remains inconsistent
Recent standardization and dosing approaches [6,8].	Platelet dose; platelet recovery; cellular composition; leukocyte/RBC content; preparation variability; activation and handling conditions	Emphasize quantification, reproducibility, and the need to move beyond the generic term “PRP”	Lack of a unified multidimensional framework integrating biological characterization with clinical and procedural variables

Abbreviations: PRP, platelet-rich plasma; RBC, red blood cells; WBC, white blood cells.

**Table 2:** Major growth factors in PRP and their role in tendon healing.

Growth Factor	Main Cellular Source	Principal Biological Functions	Predominant Phase of Tendon Healing	Potential Clinical Relevance	Key References
PDGF (Platelet-Derived Growth Factor)	Platelet α-granules; activated platelets	Chemotaxis, tenocyte and fibroblast proliferation, collagen synthesis, extracellular matrix (ECM) remodeling, angiogenesis	Early inflammatory and proliferative phases	Promotes cellular recruitment and initiation of tendon repair; may enhance matrix formation and tissue regeneration	[24,25]
TGF-β1 (Transforming Growth Factor-β1)	Platelets, macrophages, tendon cells	ECM synthesis, collagen deposition, tenogenic differentiation, immune modulation; excessive activity may promote fibrosis and scar formation	Inflammatory, proliferative, and remodeling phases	Supports matrix maturation and tendon remodeling; excessive signaling may contribute to fibrosis, adhesions, and inferior tissue quality	[24,25]
VEGF (Vascular Endothelial Growth Factor)	Platelets, endothelial cells, inflammatory cells	Angiogenesis, vascular permeability, endothelial cell proliferation, neovascularization	Early inflammatory and proliferative phases; persists during remodeling	Improves vascular supply and nutrient delivery to healing tendon; excessive neovascularization may be associated with chronic tendinopathy	[24,25]
IGF-1 (Insulin-Like Growth Factor-1)	Platelets/plasma, inflammatory cells, tenocytes, fibroblasts	Cell proliferation, protein synthesis, collagen and proteoglycan production, matrix remodeling	Early inflammatory, proliferative, and remodeling phases	Enhances collagen synthesis, tendon adaptation, structural repair, and tissue maturation; may improve biomechanical properties of healing tendon	[24,25]
bFGF (Fibroblast Growth Factor-2)	Platelets, fibroblasts, tenocytes, endothelial cells	Cell proliferation, migration, angiogenesis, collagen synthesis, ECM remodeling	Early inflammatory and proliferative phases	Stimulates early tissue regeneration and neovascularization; may accelerate tendon healing and improve matrix organization in experimental models	[24,25]

Abbreviations: PDGF, platelet-derived growth factor; TGF-β, transforming growth factor beta; VEGF, vascular endothelial growth factor; IGF-1, insulin-like growth factor-1; bFGF, fibroblast growth factor-2; bFGF, basic fibroblast growth factor; ECM, extracellular matrix; PRP, platelet-rich plasma.

**Table 3:** Predominant biological roles of the principal PRP-derived growth factors during the sequential phases of tendon healing. The table summarizes the temporal predominance of PDGF, TGF-β1, VEGF, IGF-1, and bFGF across the inflammatory, proliferative, and remodeling phases, together with their principal cellular targets and biological functions.

Healing Phase	Dominant Mediators	Principal Biologic Events
Inflammatory	PDGF, TGF-β1	Chemotaxis, inflammation
Proliferative	TGF-β, VEGF	Angiogenesis, ECM synthesis
Remodeling	IGF-1, bFGF	Collagen maturation

**Table 4:** Proposed operational Q-PRP reporting protocol, comprising core/minimum and extended/research variables for standardized characterization of PRP in tendon studies.

Domain	Variable	Reporting Requirement	Recommended Descriptor/Unit	Q-PRP Level	Key References
Cellular	Baseline whole-blood platelet count	Report measured value before PRP preparation	Platelets/µL	Core	[6,8,50]
Cellular	Final PRP platelet concentration	Report measured concentration in the administered product	Platelets/µL	Core	[6,10,50]
Cellular	Total platelet dose delivered	Calculate from final platelet concentration × administered PRP volume	Total platelets, preferably ×10^9^	Core	[5,10,11,12]
Cellular	Total leukocyte concentration	Report measured value in the final PRP product	Cells/µL	Core	[5,6,8,13]
Cellular	Leukocyte differential	Report neutrophil, lymphocyte and monocyte proportions/counts when available	% and/or cells/µL	Core *	[6,8,13,14,15,16]
Cellular	RBC contamination	Quantify residual erythrocytes in the final PRP product	Cells/µL, hematocrit %, or validated equivalent	Core	[5,8,10]
Cellular	Platelet recovery efficiency	Report the proportion of baseline platelets recovered when calculable	%	Extended	[8,9,10]
Molecular	Growth factor profile	Quantify selected mediators relevant to the study hypothesis (e.g., PDGF, TGF-β, VEGF, IGF-1, FGF-2)	Concentration per mL; assay specified	Extended	[12,22,23,24]
Molecular	Cytokine profile	Quantify selected inflammatory mediators when mechanistically relevant (e.g., IL-1β, IL-6, TNF-α, IL-10)	Concentration per mL; assay specified	Extended	[12,15,16,31]
Procedural	Whole-blood draw volume	Report total blood volume collected for PRP preparation	mL	Core	[8,42,51]
Procedural	Anticoagulant	Report anticoagulant type and, where applicable, concentration or blood: anticoagulant ratio	Agent; concentration/ratio	Core	[8,42,51]
Procedural	Centrifugation protocol	Report the number of spins and complete centrifugation parameters	RCF (×g), duration, number of spins	Core	[8,9,42,51]
Procedural	Final PRP volume	Report final volume obtained and volume actually administered	mL	Core	[8,10,42,51]
Procedural	Activation strategy	Report whether PRP was non-activated/endogenously activated or exogenously activated; specify activator and protocol	Method; agent; concentration	Core	[6,8,10,42,51]
Procedural	Time from preparation to administration	Report interval from preparation/activation to administration when applicable	Minutes	Core	[8,42,51]
Procedural	Injection number and schedule	Report number of administrations and interval between treatments	Number; days/weeks	Core	[3,5,8,42]
Procedural	Delivery technique	Report anatomical target and whether imaging guidance was used	Intratendinous/peritendinous/enthesis; image-guided yes/no	Core	[3,8,42,51]
Clinical	Tendon disorder and anatomical site	Define the treated tendon disorder and anatomical location	Standardized clinical diagnosis	Core	[3,4,5,8]
Clinical	Disease stage/chronicity	Report symptom duration and structural severity when available	Weeks/months; validated grading system if used	Core	[3,4,8,43]
Clinical	Patient characteristics	Report major recipient factors potentially affecting PRP biology or clinical response	Age, sex, relevant comorbidities, smoking status, relevant medications	Core	[4,5,8,42]
Clinical	Rehabilitation protocol	Describe post-treatment loading/exercise sufficiently to permit replication	Timing, type and progression	Core	[4,5,8,42]
Clinical	Clinical outcome measures	Use validated tendon-specific and/or functional outcome measures	E.g., VISA-P/VISA-A, DASH/PRTEE, pain scales	Core	[3,4,5,8]
Clinical	Follow-up	Report predefined assessment intervals and total follow-up	Weeks/months	Core	[3,4,5,8]
Clinical	Imaging biomarkers	Include objective imaging outcomes when incorporated into the study design	US/Doppler/elastography/MRI; quantitative metric specified	Extended	[41,49]
Clinical/Precision	Molecular/genetic patient modifiers	Report when specifically investigated as potential predictors of biological or clinical response	Genotype/SNP; analytical method	Extended	[52,53,54,55]

* if only total leukocyte concentration is technically available, this should be explicitly stated; differential leukocyte characterization is preferred because neutrophils, monocytes, and lymphocytes have distinct biological effects.

## Data Availability

No new data were created or analyzed in this study. Data sharing is not applicable to this article.

## Abbreviations

The following abbreviations are used in this manuscript:

ADP	Adenosine diphosphate
AI	Artificial intelligence
ATP	Adenosine triphosphate
bFGF	Basic fibroblast growth factor
CaCl_2_	Calcium chloride
DASH	Disabilities of the arm, shoulder and hand
DEPA	Dose, efficiency, purity, activation (PRP classification system)
ECM	Extracellular matrix
EGF	Epidermal growth factor
ERK	Extracellular signal-regulated kinase
HGF	Hepatocyte growth factor
HIF-1α	Hypoxia-inducible factor-1 alpha
IGF-1	Insulin-like growth factor-1
IL-1β	Interleukin-1 beta
IL-6	Interleukin-6
IL-10	Interleukin-10
JAK	Janus kinase
LP-PRP	Leukocyte-poor platelet-rich plasma
LR-PRP	Leukocyte-rich platelet-rich plasma
MAPK	Mitogen-activated protein kinase
MARSPILL	Method, activation, red blood cells, spin, platelets, image guidance, leukocytes, light activation
MIBO	Minimum information for studies evaluating biologics in orthopedics
MMP	Matrix metalloproteinases
MRI	Magnetic resonance imaging
NF-κB	Nuclear factor kappa B
NSAIDs	Non-steroidal anti-inflammatory drugs
PAR-1	Protease-activated receptor-1
PAR-4	Protease-activated receptor-4
PAW	Platelets, activation, white blood cells (PRP classification system)
PDGF	Platelet-derived growth factor
PDGF-BB	Platelet-derived growth factor- BB
PDGFR	Platelet-derived growth factor receptor
PDGFRA	Platelet-derived growth factor receptor alpha gene
PDGFRB	Platelet-derived growth factor receptor beta gene
PI3K	Phosphoinositide 3-kinase
PRP	Platelet-rich plasma
Q-PRP	Quantifiable platelet-rich plasma
RBC	Red blood cells
RNA	Ribonucleic acid
ROCK	Rho-associated coiled-coil containing protein kinase
SMAD	Mothers against decapentaplegic homolog proteins
SNP	Single-nucleotide polymorphism
STAT	Signal transducer and activator of transcription
TGF-β	Transforming growth factor-beta
TNF-α	Tumor necrosis factor-alpha
UTE	Ultrashort echo time
VEGF	Vascular endothelial growth factor
VEGFA	Vascular endothelial growth factor A gene
VEGFR-2	Vascular endothelial growth factor receptor-2
VISA-A	Victorian Institute of Sport Assessment–Achilles
VISA-P	Victorian Institute of Sport Assessment–Patellar
WBC	White blood cells
